# Behavioral and computational perspectives on emotion regulation and subjective wellbeing: an integrative review with a focus on adolescents

**DOI:** 10.3389/fpsyg.2026.1778913

**Published:** 2026-09-16

**Authors:** Libin Jiang, Luying Yi

**Affiliations:** School of Education and Psychology, Minnan Normal University, Zhangzhou, Fujian, China

**Keywords:** adolescents, affective computing, computational modeling, digital phenotyping, emotion regulation, empirical synthesis, mental health, self-esteem

## Abstract

Understanding how emotional regulation (ER) mediates the relationship between self-esteem and subjective wellbeing (SWB) is central to promoting mental health among high school students. This paper provides a comprehensive integrative review of theoretical and empirical research exploring this interplay, while integrating perspectives from behavioral sciences and computational psychology. Traditional research primarily relies on self-report measures and cross-sectional designs, which limit the detection of dynamic emotional processes. On the other hand, emerging computational methods, such as machine learning-based mediation modeling and linguistic sentiment analysis enable new approaches to quantify and predict ER and wellbeing. This review develops an integrative framework that connects behavioral constructs and empirical evidence on adolescent ER and SWB with computational approaches for modeling these relationships. Rather than treating behavioral and computational methods as separate approaches, the framework considers how psychological measures, contextual and digital data, computational modeling, and adaptive feedback can be combined to support a more comprehensive understanding of adolescent wellbeing.

## 1 Introduction

Adolescence represents a critical period of development characterized by profound biological and cognitive changes that collectively shape an individual's sense of self and psychological adjustment. During this phase, young people experience high emotional reactivity and a growing need for autonomy and social belonging (Steinberg, 2005; Casey et al., 2008). The ability to manage emotional experiences adaptively, referred to as *emotional regulation* (ER), plays a central role in determining mental health and subjective wellbeing (SWB). ER refers to the processes through which individuals modulate their emotional responses in relation to their goals and situational demands. Importantly, ER is not inherently adaptive or maladaptive. The effectiveness of a regulatory response depends on the emotion being regulated, the individual's goals and the demands of the situation (Gross and Jazaieri, 2014; Springstein and English, 2024; McCullen and Scott, 2024). ER not only buffers against anxiety, depression and stress but also enhances resilience, empathy, and interpersonal competence (Gross, 2015; Serey et al., 2025).

SWB, defined by Diener (1984) as the cognitive and affective evaluation of one's life, is a multidimensional construct encompassing both positive and negative emotional experiences. It is influenced by internal factors, such as self-esteem, EI and personality, as well as external factors, such as family and peer support and the environment of the school in teenagers (King et al., 2023; Aldridge and McChesney, 2018). Adolescents with higher levels of wellbeing demonstrate greater academic achievement and healthier social relationships (Hernández-Torrano et al., 2025; Tejada-Gallardo et al., 2020). On the other hand, poor ER and low self-esteem lead to psychological distress and academic disengagement (Goldin and Gross, 2010; Mendes and Miguel, 2024).

Self-esteem can be understood from complementary perspectives that emphasize both an individual's general evaluation of the self and the extent to which this evaluation is shaped by social and contextual experiences. Although higher self-esteem is generally associated with more favorable psychological outcomes, the relationship is not necessarily uniform across individuals or situations. Social feedback, perceived acceptance, competence, and interpersonal experiences can influence how self-evaluations are formed and maintained, making self-esteem particularly relevant to adolescent wellbeing. This contextual perspective is important for the present framework because computational approaches may capture behavioral or contextual indicators that complement conventional self-report measures of self-esteem.

Over the past two decades, research in psychology and psychiatry has increasingly emphasized the role of ER as a *mediator* between individual traits, e.g., self-esteem, EI and psychological outcomes such as wellbeing, anxiety or depression (Gross, 2015; Berking and Wupperman, 2012). The mediating influence of ER suggests that how adolescents manage emotions is as important as what emotions they experience. For example, cognitive reappraisal, i.e., the process of reinterpreting an emotional event to change its impact, has been linked to higher wellbeing and fewer internalizing symptoms, whereas maladaptive strategies like rumination and suppression predict poorer outcomes (Lai, 2024).

Importantly, understanding adolescent wellbeing requires consideration not only of the emotions adolescents experience but also of how they regulate those emotions and the specific strategies they employ. The consequences of an emotion-regulation strategy depend on the individual's goals and the social and situational context in which it is used; therefore, strategies should not be characterized as inherently adaptive or maladaptive (McCullen and Scott, 2024; Springstein and English, 2024). A strategy that is useful in one context may be less effective, or even counterproductive, in another. This perspective highlights the importance of measuring specific ER strategies and their contextual effectiveness rather than relying solely on global indicators of ER or dysregulation.

Within educational contexts, schools have emerged as vital settings for fostering emotional competence and wellbeing. Programs rooted in mindfulness and social-emotional learning (SEL) have shown effectiveness in promoting adaptive ER and mental health (Broderick and Jennings, 2012; Kennes et al., 2023; DeBiase et al., 2021; Razza et al., 2025). For instance, school-based interventions combining mindfulness and character strengths have improved students' emotional awareness and life satisfaction (Kennes et al., 2023; Tejada-Gallardo et al., 2020). Similarly, meta-analyses of positive psychology interventions indicate consistent increases in wellbeing and reductions in psychological distress among adolescents (Tejada-Gallardo et al., 2020; Hernández-Torrano et al., 2025).

Despite these advances, several limitations persist in existing literature. Most studies rely heavily on self-report surveys and cross-sectional designs, limiting the ability to capture the dynamic, contextual and temporal nature of ER. Moreover, cultural variations in emotional expression and wellbeing are often overlooked, which leads to a Western-centric bias in theoretical models and measurement tools (King et al., 2023; Schouwenaars and Gravesteijn, 2025). There is also limited integration between psychological theory and computational techniques capable of uncovering complex interactions between emotion and behavior.

Recent developments in computational psychiatry and affective computing offer new methodological possibilities for addressing these gaps (Montague et al., 2012; Picard, 1997; Calvo and D'Mello, 2010). Through machine learning (ML) and natural language processing (NLP), researchers can now model ER and wellbeing with greater precision and ecological validity (Jiang et al., 2021; Schek et al., 2017). For example, digital phenotyping, using wearable sensors or social media data, enables continuous monitoring of emotional states, while mediation models powered by ML can reveal nonlinear interactions between variables traditionally treated as linear (Khaleghi et al., 2022). Moreover, positive computing–technologies designed to support wellbeing has been increasingly applied to adolescent populations to enhance emotional literacy and coping (Schek et al., 2017; Calvo and D'Mello, 2010).

The integration of computational approaches with behavioral science represents a paradigm shift toward *data-informed mental health*. By coupling traditional psychometric assessments with digital and physiological measures, it becomes possible to build predictive models of adolescent wellbeing that are culturally sensitive and dynamic. Such an interdisciplinary perspective aligns with the global movement toward precision psychiatry and personalized interventions (Friston, 2023).

Although the behavioral and computational framework presented in this paper is sufficiently general to be adapted to other age groups, adolescence is intentionally adopted as the primary application context. This developmental period is marked by substantial biological, cognitive, emotional, and social transitions that profoundly influence self-esteem, ER, and SWB. These characteristics make adolescence an especially informative stage for studying the interactions among these constructs and for evaluating computational approaches that aim to monitor and promote mental wellbeing. Moreover, recent advances in digital mental health and educational technologies have generated a rapidly growing body of adolescent-focused research, providing a strong empirical foundation for the present review.

Accordingly, this review has four objectives: (1) To synthesize behavioral and psychological evidence concerning ER and SWB in adolescence. (2) To examine how computational approaches can complement established behavioral methods for studying these constructs. (3) To identify empirical and methodological trends that support integration of behavioral and computational perspectives. (4) To propose an integrative framework linking psychological constructs, multimodal data, computational modeling, and adaptive feedback while considering ethical and contextual constraints.

The primary contribution of this review is an integrative framework that brings together behavioral theory, empirical evidence and computational approaches to examine adolescent emotion regulation and subjective wellbeing. The framework does not propose a new predictive algorithm or a new psychological theory. Instead, it provides a conceptual and methodological bridge between established psychological constructs and emerging computational methods. This allows us to illustrate how behavioral measures, contextual and digital data, computational modeling, and adaptive feedback can be considered within a common research architecture. In this sense, the manuscript positions computational approaches as complementary to, rather than substitutes for, behavioral theory and measurement.

Although adolescent wellbeing is influenced by numerous psychological and social factors. They usually include resilience, anxiety, depression, self-efficacy, family functioning, and social support. However, this paper deliberately focuses on ER and SWB because these constructs constitute one of the most extensively validated theoretical pathways in developmental and positive psychology. ER captures the mechanisms through which emotions are managed and adapted. Finally, SWB reflects the overall psychological outcome encompassing life satisfaction and affective functioning. Moreover, these constructs have been consistently operationalized using validated psychometric instruments and have become the primary targets of recent computational modeling efforts. This enables meaningful integration of behavioral theories with machine learning and digital phenotyping approaches. By concentrating on this theoretically coherent triad, the paper provides a focused synthesis while recognizing that other determinants of adolescent mental health remain important directions for future investigation.

### 1.1 Review approach

This study adopts a narrative and integrative review approach to synthesize evidence at the intersection of adolescent psychology, emotion regulation, subjective wellbeing, computational modeling, and artificial intelligence. Narrative reviews are particularly appropriate when the literature spans multiple disciplines and includes heterogeneous theoretical perspectives, methodologies, and forms of evidence that cannot readily be combined through a single quantitative synthesis (Green et al., 2006; Baumeister and Leary, 1997). The objective was therefore not to provide an exhaustive systematic review or meta-analysis, but to develop a theoretically informed synthesis linking established psychological constructs with emerging computational approaches.

The literature search was conducted across major scholarly databases and search platforms, including Scopus, Web of Science, PubMed, Google Scholar, and IEEE Xplore, with emphasis on literature published from approximately 2000 to 2025 and additional seminal and recent studies identified through reference-list screening. The search strategy combined terms relating to the principal psychological constructs and the computational dimension of the review. Representative combinations included “emotion regulation” AND “subjective wellbeing,” “self-esteem” AND “emotion regulation” AND adolescents, “emotion regulation” AND adolescents AND “wellbeing,” “computational psychiatry” AND emotion, “artificial intelligence” AND adolescent mental health, “machine learning” AND emotion regulation, “digital phenotyping” AND wellbeing, and “AI” AND student wellbeing. Additional searches were conducted using closely related terms when emerging computational or intervention literature used different terminology.

Literature was considered relevant when it contributed directly to one or more of the central constructs or relationships examined in the review, including self-esteem, emotion regulation, subjective wellbeing, adolescent development, computational modeling, digital phenotyping, artificial intelligence, or technology-assisted psychological intervention. Priority was given to peer-reviewed empirical studies, theoretically influential publications, methodological contributions, systematic or meta-analytic evidence where relevant, and recent computational studies that could inform the proposed interdisciplinary framework. Studies were also considered when they provided important conceptual or methodological evidence from adjacent populations where direct adolescent evidence remained limited. Studies that were primarily unrelated to the central constructs, lacked sufficient relevance to the psychological or computational focus of the review, or did not contribute to the development of the integrative argument were not emphasized.

The identified literature was organized thematically rather than treated as a single homogeneous evidence base. First, psychological literature was examined to establish the conceptual relationships among self-esteem, emotion regulation, and subjective wellbeing. Second, traditional empirical approaches and commonly used measurement instruments were reviewed to identify established patterns and methodological limitations. Third, computational and technology-enhanced approaches were examined, including machine learning, multimodal data, digital phenotyping, and computational approaches to psychological assessment. Fourth, empirical trends were compared across behavioral and computational studies to identify areas of convergence, inconsistency, and methodological gaps. Finally, the findings were synthesized into an integrative behavioral-computational framework that connects psychological constructs, computational modeling, and context-sensitive feedback.

The synthesis therefore emphasized conceptual convergence and methodological complementarity rather than numerical aggregation of effect sizes. Particular attention was given to whether computational approaches preserve theoretically meaningful psychological constructs, whether they provide additional temporal or contextual information beyond conventional self-report measures, and how computational findings could inform ethically grounded and personalized approaches to adolescent wellbeing. Studies described as “selected” or “representative” in the tables were those identified as particularly relevant to these theoretical, empirical, or methodological objectives; these labels do not imply an exhaustive or systematic selection of all available studies.

### 1.2 Organization of the paper

The rest of this paper is organized as follows: Section 2 presents the *Conceptual Framework*, discussing key constructs, i.e., ER, SWB and self-esteem, and explaining their theoretical interrelations. Section 3 reviews *Traditional Empirical Approaches*, highlighting measurement tools, psychometric models and the limitations of survey-based research. Section 4 introduces the integration of computational psychiatry and affective computing, showing how artificial intelligence, natural language processing and machine learning can model emotional processes. Section 5 synthesizes empirical findings and trends, comparing behavioral and computational insights. Section 6 discusses key challenges and ethical considerations, while proposing future interdisciplinary research in this area. Finally, Section 7 concludes the paper.

## 2 Conceptual framework

### 2.1 Definitions and core constructs

This review focuses on three core psychological constructs and their theoretical interrelations: SWB, ER and self-related attributes, such as *self-esteem* and *emotional intelligence* (EI).

#### 2.1.1 Subjective wellbeing

Diener's seminal work defines SWB as the cognitive and affective evaluation of one's life, comprising life satisfaction and the relative absence of negative affect (Diener, 1984). SWB is influenced by stable dispositional traits, e.g., personality, contextual elements, such as family and school climate, and dynamic regulatory processes. High SWB in adolescents correlates with improved academic performance and social adjustment (Hernández-Torrano et al., 2025; Aldridge and McChesney, 2018).

SWB is likewise multidimensional and extends beyond a single indicator of positive mood. It commonly encompasses individuals' evaluations of their lives as well as their experiences of positive and negative affect. This distinction is relevant to the present framework because computational indicators may capture momentary or contextual changes in affect, whereas conventional wellbeing measures can provide a broader assessment of individuals' overall evaluations of their lives. Combining these perspectives may therefore provide a more comprehensive representation of adolescent wellbeing.

#### 2.1.2 Emotional regulation

ER refers to the processes by which individuals influence which emotions they have and how they experience and express these emotions (Gross, 1998, 2015). Gross (1998) process model classifies regulatory strategies into antecedent-focused, e.g., cognitive reappraisal, and response-focused, e.g., expressive suppression, and demonstrates differential associations with psychological outcomes. Adaptive strategies, such as reappraisal, are consistently linked to better wellbeing, whereas suppression and rumination relate to poorer outcomes (Lai, 2024).

ER can therefore be viewed not simply as the presence or absence of regulation, but as a context-dependent process in which individuals modify emotional responses in relation to their goals and situational demands. This perspective also highlights the importance of considering specific regulation strategies and their consequences rather than assuming that particular strategies are inherently adaptive or maladaptive. Such distinctions are important when translating psychological constructs into computational features. This is because the same observable behavior may have different meanings depending on emotional and contextual circumstances.

#### 2.1.3 Self-esteem and emotional intelligence

Self-esteem is the evaluative component of self-concept and has been robustly associated with adolescent wellbeing (Rosenberg, 1965; Mendes and Miguel, 2024). Emotional intelligence (EI), originally conceptualized by Mayer and Salovey, denotes the ability to perceive, use, understand and manage emotions. A higher EI predicts more adaptive regulation and higher SWB (Mayer and Salovey, 1990; Vieira and Castro, 2021).

### 2.2 Mediated relationships: ER as the mechanism

A growing body of adolescent psychology research supports the idea that ER functions as a mediator between dispositional traits, such as self-esteem or EI, and outcomes related to SWB. As shown in Figure 1, this mediated pathway typically includes both an indirect and a direct component. The indirect pathway captures the process through which self-related resources enhance regulatory capacity, which in turn increases wellbeing, whereas the direct pathway captures the portion of influence that operates independently of ER (Preacher and Hayes, 2008; Berking and Wupperman, 2012; Jiang et al., 2021). It is important to note that the model in Figure 1 represents one component of the broader behavioral-computational perspective developed in this paper, rather than the overarching framework of the paper.

**Figure 1:**
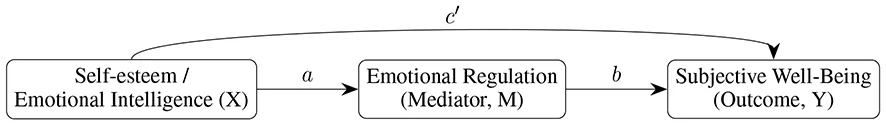
Conceptual mediation model illustrating the indirect effect of self-esteem or emotional intelligence on subjective wellbeing through ER (*a* × *b*), alongside a direct effect (*c*′).

Empirical studies among adolescents have generally shown that self-esteem and EI predict better ER, which in turn partly explains higher life satisfaction and lower psychological distress. This indicates that emotion regulation acts as a partial mediator between these dispositional resources and SWB, although the strength of this effect and the prominence of specific strategies, such as cognitive reappraisal or suppression, vary across cultural contexts, gender, and exposure to emotional skills interventions (Mendes and Miguel, 2024; Serey et al., 2025; Koçak and Terzi, 2023). Meta-analytic evidence confirms that adaptive antecedent-focused strategies, e.g., reappraisal, are more strongly associated with positive affect and life satisfaction than response-focused strategies, e.g., suppression (Berking and Wupperman, 2012). These findings emphasize the need to examine mediating mechanisms rather than direct correlations alone when assessing adolescent mental health.

### 2.3 Theoretical grounding: process, social-cognitive and developmental perspectives

The proposed mediated relationship is grounded in several complementary theoretical frameworks. The first is the *process model of emotion regulation*, developed by Gross (1998, 2015), which distinguishes between antecedent-focused and response-focused strategies, highlighting that adaptive strategies, such as cognitive reappraisal are generally linked with higher SWB and resilience.

The second is *Bandura's social-cognitive theory*, which explains how self-efficacy beliefs shape behavioral regulation and emotional outcomes. Adolescents who believe in their ability to manage emotional challenges are more likely to employ adaptive strategies and consequently experience greater wellbeing (Bandura, 1977, 1997).

Finally, a *developmental-contextual perspective* recognizes that adolescence is marked by neurobiological changes in the prefrontal cortex and limbic system, as well as evolving social dynamics. These developmental transitions influence both the emergence and sophistication of ER strategies (Casey et al., 2008; Steinberg, 2005). Integrating these perspectives offers a holistic understanding of how ER functions as a developmental bridge between self-related resources and psychological wellbeing.

### 2.4 Computational perspective on mediated analysis

Traditional mediation analysis, typically conducted through regression-based or structural equation modeling (SEM) frameworks, assumes linear and static relationships. In contrast, computational approaches in psychiatry and affective science allow researchers to model dynamic and individualized pathways between psychological constructs.

As visualized in Figure 2, computational extensions contribute at three levels: (1) Multimodal measurement in which wearable sensors, smartphone usage logs and social media data enable continuous ecological assessment of emotion and behavior. This is often referred to as *digital phenotyping* (Picard, 1997; Calvo and D'Mello, 2010). (2) Flexible modeling, which involves machine learning techniques, such as random forests or neural networks, that can detect complex nonlinear mediation effects and interactions that traditional SEM cannot capture (Montague et al., 2012; Jiang et al., 2021). (3) Real-time intervention, which involves models that can inform *just-in-time adaptive interventions* (JITAIs) by dynamically adjusting mindfulness or counseling prompts based on the individual's emotional state (Schek et al., 2017).

**Figure 2:**
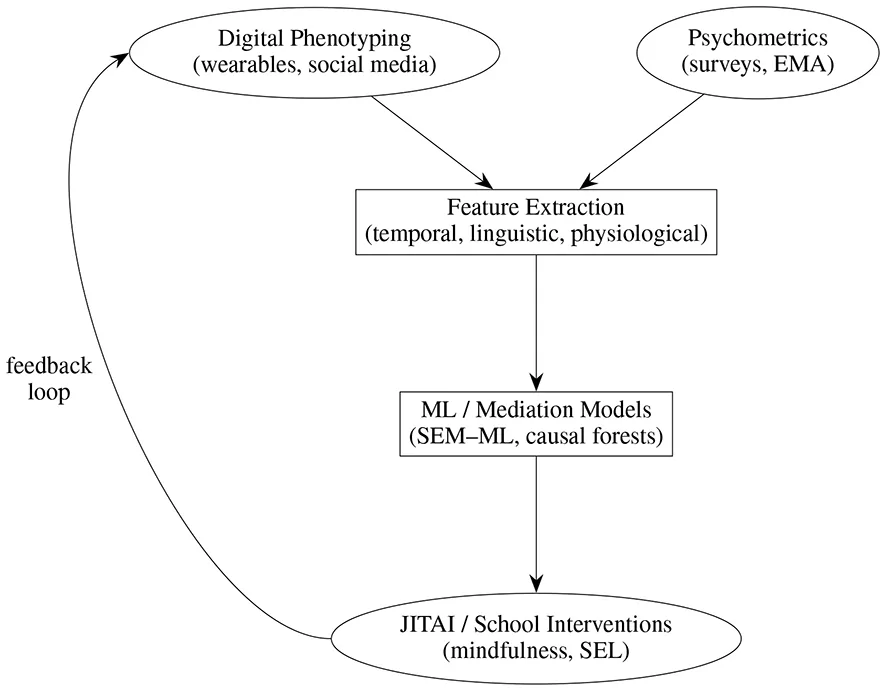
Computational integration of digital phenotyping and psychometrics into mediation models that inform JITAIs for adolescent wellbeing. The feedback loop illustrates continuous model refinement and outcome evaluation.

By embedding psychological mediation frameworks within computational psychiatry, future studies can move beyond static correlations toward personalized, predictive models of emotional functioning. This hybrid perspective, i.e., linking behavioral science with data-driven inference, sets the stage for the methodological discussion in Section 3 and the computational analyses in Section 4.

## 3 Traditional empirical approaches

Traditional empirical research on ER, self-esteem and SWB in adolescents has primarily relied on psychometric assessment and correlational designs as shown in Table 1. This section synthesizes key measurement tools and empirical findings in the field, drawing on over two decades of research across diverse cultural contexts.

**Table 1:** Selected empirical studies on ER and SWB in adolescents.

Measurement tools	Population/context	Key findings	References
ERQ, life satisfaction scale	Italian high school students	Cognitive reappraisal positively predicted wellbeing; expressive suppression associated with lower psychological health.	Verzeletti et al., 2016
Ryff's PWBS	Spanish adolescents	Psychological wellbeing correlated with adaptive ER and social support; scale shown valid cross-culturally.	Molina Moreno et al., 2023
Regulation of emotions questionnaire (REQ)	UK adolescents	Introduced adolescent-specific measure for functional vs. dysfunctional ER; reliability confirmed.	Phillips and Power, 2007
ER ability tests, SHS, school satisfaction	U.S. adolescents	Performance-based and observer-reported ER independently predicted relationship quality and school success.	St-Louis et al., 2021
DERS, EPOCH, RSA (physiological)	Australian adolescents	Combined physiological and self-report markers; RSA linked to adaptive regulation and wellbeing.	Morrish et al., 2019
Review of ER scales	Global review	Identified methodological inconsistencies; highlighted few tools validated.	Mazefsky et al., 2021
ERQ, SWLS, SPANE	Indonesian adolescents	No significant correlation between ER and SWB found; highlights contextual variability and measurement bias.	Widiastuti and Widyastuti, 2023

### 3.1 Measurement instruments and constructs

The most widely adopted tools for assessing ER include the *Emotion Regulation Questionnaire* (ERQ) (Gross, 1998), the *Difficulties in Emotion Regulation Scale* (DERS) (Gratz and Roemer, 2004) and the *Trait Meta-Mood Scale* (TMMS) (Salovey et al., 1995). These self-report instruments emphasize distinct regulatory dimensions, cognitive reappraisal and expressive suppression can be done via ERQ, difficulties in managing emotions via DERS and meta-cognitive awareness of mood states using TMMS. Despite their popularity, these scales were originally developed for adults, which raises validity concerns when applied to adolescent populations (Phillips and Power, 2007).

Although much of the existing ER literature has emphasized the down-regulation of negative emotions, regulation of positive emotions is also relevant to adolescent wellbeing. Adolescents may seek to maintain or enhance positive emotions, but may also regulate their intensity depending on social and situational goals. Recent work by McCullen and Scott (2024), for example, considers reappraisal and suppression in the regulation of positive emotions, highlighting an aspect of adolescent ER that has received comparatively less attention. Including both positive and negative ER therefore provides a more comprehensive basis for understanding how specific regulatory strategies relate to subjective wellbeing.

For SWB, the most frequently employed instruments include the *Satisfaction with Life Scale* (SWLS), the *Subjective Happiness Scale* (SHS), the *EPOCH Measure of Adolescent Wellbeing*, and Ryff's *Psychological Wellbeing Scales* (PWBS). These measures tap into both hedonic (life satisfaction, happiness) and eudemonics (purpose, autonomy) dimensions of wellbeing (Molina Moreno et al., 2023; Salazar and Warren, 2024). The continued adaptation and validation of these instruments across cultural settings highlight the growing international recognition of adolescent SWB as a developmental priority.

### 3.2 Methodological limitations and cultural considerations

Most studies employ self-report Likert-type scales administered through cross-sectional surveys, which are limited by social desirability and recall bias. Moreover, the overreliance on Western-derived instruments can obscure contextually specific expressions of wellbeing or ER, especially in collectivist cultures (Molina Moreno et al., 2023). As noted in Figure 1, the mediating role of ER is often inferred statistically rather than measured through multi-modal behavioral data.

Cultural adaptations, such as those conducted in Indonesia and Iran, demonstrate the need for psychometric validation that captures local constructs of emotional maturity, family support and gratitude (Keshavarz et al., 2025; Biao and Xinmei, 2015). The increasing use of Rasch modeling and item response theory (IRT) for instrument refinement has improved measurement precision and construct validity in recent years (Ireland et al., 2018).

### 3.3 Emerging mixed-method and physiological approaches

Recent empirical work has sought to bridge subjective and objective measures of ER and SWB through physiological monitoring, behavioral observation and ecological momentary assessment (EMA). The feasibility of combining passive audio sampling via the *Electronically Activated Recorder* (EAR) has been demonstrated with EMA surveys to track emotion regulation in real-life contexts (Kaplan et al., 2022). Similarly, integrated heart rate variability indices have been integrated as objective markers of regulatory capacity. These hybrid designs mark a gradual transition toward the computational integration described in Figure 3.

**Figure 3:**
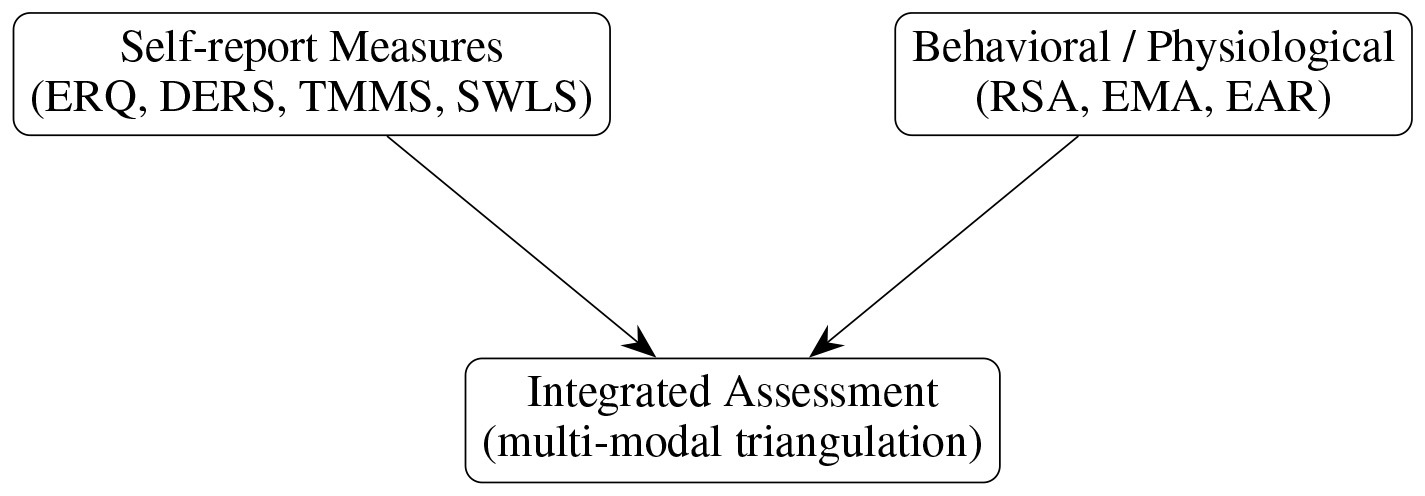
Transition from traditional self-report to integrated multimodal approaches in emotion regulation and wellbeing assessment.

### 3.4 Synthesis and implications

Traditional empirical studies have established strong correlational links between ER and SWB but remain limited in causal inference and ecological validity. The dominant reliance on cross-sectional self-reports restricts understanding of dynamic processes, while the lack of multi-level data (e.g., neural, physiological, behavioral) constrains the ability of generalizing the model.

The emerging research trajectory points toward a hybrid paradigm that integrates psychometric rigor with computational modeling and real-time data collection. Such integration, outlined in Section 4, holds promise for uncovering nuanced, context-sensitive pathways between ER and SWB in adolescents.

### 3.5 Discussion of empirical synthesis

Table 2 summarizes key self-report instruments used to assess ER and SWB among adolescents. These instruments vary in their theoretical underpinnings, i.e., from Gross's process model of ER to Diener's conceptualization of life satisfaction, and demonstrate wide psychometric application across contexts.

**Table 2:** Common instruments used to assess ER and SWB in adolescents.

Instrument	Construct(s) measured	No. of items/subscales	Primary relevance to the review	Evidence type
ER questionnaire (ERQ)	Cognitive reappraisal and expressive suppression	10 items/two subscales	Assesses commonly studied emotion-regulation strategies and supports comparison of strategy use across adolescent studies (e.g., Gross, 1998).	Empirical measure
Difficulties in ER scale (DERS)	Difficulties in ER, including impulse control, emotional awareness, and access to regulation strategies	36 items/six subscales	Provides a multidimensional assessment of difficulties in regulating emotions and complements strategy-focused measures such as the ERQ (e.g., Gratz and Roemer, 2004).	Empirical measure
Trait meta-mood scale (TMMS-24)	Emotional attention, emotional clarity, and emotional repair	24 items/three subscales	Assesses perceived awareness and understanding of emotional states and the perceived ability to regulate mood (e.g., Salovey et al., 1995).	Empirical measure
Satisfaction with life scale (SWLS)	Cognitive evaluation of global life satisfaction	Five items/single factor	Provides a widely used measure of the cognitive component of SWB and is frequently examined alongside ER (e.g., Diener et al., 1985).	Empirical measure
EPOCH measure of adolescent wellbeing	Engagement, perseverance, optimism, connectedness, and happiness	20 items/five subscales	Captures multiple positive dimensions of adolescent wellbeing and extends assessment beyond global life satisfaction (e.g., Kern et al., 2016).	Adolescent-specific measure
Ryff's psychological wellbeing scales (PWBS)	Autonomy, purpose in life, self-acceptance, and environmental mastery, among other dimensions	42 items/six subscales	Represents a eudaimonic perspective on wellbeing, complementing predominantly hedonic measures of SWB (e.g., Ryff, 1989).	Empirical measure
Subjective happiness scale (SHS)	Global subjective judgments of happiness	Four items/single factor	Provides a brief global indicator of subjective happiness that can complement multidimensional wellbeing measures (e.g., Lyubomirsky and Lepper, 1999).	Empirical measure

Across the reviewed works, ER emerges as both a predictor and a mediator of adolescent wellbeing. For example, it has been shown that cognitive reappraisal enhances wellbeing and social relationships, while expressive suppression tends to predict maladaptive outcomes (Verzeletti et al., 2016). Similarly, psychological wellbeing has been found to be significantly related to adaptive emotion regulation and perceived social support in Spanish youth (Molina Moreno et al., 2023). However, studies like Widiastuti and Widyastuti (2023) report inconsistent associations in collectivist cultures, suggesting that contextually adapted psychometrics are vital for valid inference.

Meta-analytic reviews, such as Mazefsky et al. (2021) and Serey et al. (2025), reveal persistent limitations, i.e., over reliance on self-report, absence of longitudinal validation and limited cross-cultural representation. Recent hybrid studies Kaplan et al. (2022) and Morrish et al. (2019) combining physiological and ecological data have begun addressing these gaps, yet computational integration in adolescent populations remains underexplored.

Taken together, the behavioral literature provides converging evidence that ER and self-evaluative processes are associated with adolescent SWB, while also suggesting that these relationships are influenced by contextual and individual differences. However, the studies differ substantially in their operationalization of ER, the use of self-report measures, and the extent to which potential mediating and moderating mechanisms are examined. Much of the evidence is also based on cross-sectional designs, which limits conclusions about temporal ordering and developmental change. Thus, although the literature supports the relevance of the proposed psychological constructs, it provides less clarity regarding how these relationships unfold dynamically across contexts and over time.

In conclusion, traditional empirical approaches have provided foundational insight into adolescent emotion regulation and SWB, but methodological fragmentation and limited multimodal integration constrains the generalization. Section 4 explores computational and technology-enhanced methodologies that can bridge these gaps and enable dynamic and culturally sensitive modeling.

## 4 Computational and technology-enhanced approaches

Recent advances in computational psychiatry and educational technology have enabled quantitative modeling of ER and SWB in adolescents. These approaches integrate psychometric data and behavioral signals along with digital traces, offering scalable and personalized mental-health insights (Abro et al., 2014; Jiang et al., 2021). In this section, we review key developments under three themes, i.e., data-driven ER, computational mediation modeling and human-AI collaboration, while highlighting emerging challenges.

### 4.1 Data-driven ER

Data-driven emotion research increasingly leverages multimodal information–from self, ranging from reports to smartphone sensing and social media, to infer emotion dynamics in real time. It has been demonstrated that integrating coping styles, ER and SWB through machine learning achieved predictive accuracy superior to traditional linear models (Jiang et al., 2021). Similarly, dynamic structural equation modeling (DSEM) has been used to capture day-to-day emotional variability among adolescents, illustrating how digital diaries and ecological momentary assessment (EMA) can uncover hidden regulatory patterns (Wenzel et al., 2022).

Digital phenotyping Montague et al. (2012) further extends these analyses by combining passive smartphone and wearable data with psychometric indicators. it has been highlighted in Salazar and Warren (2024) that data-driven emotion measurement can enhance construct validity when complemented by self-report tools, such as the Rosenberg Self-Esteem Scale (Rosenberg, 1965). These approaches bridge traditional psychology with computational behavioral science, supporting individualized models of emotional wellbeing.

### 4.2 Computational mediation models

Recent works employ advanced mediation and causal modeling to examine how ER mediates between self-related constructs and SWB. Psychological questionnaires have been combined with affective-computing features extracted from voice and text data, applying structural equation modeling with machine learning (SEM-ML) (Abro et al., 2014). Their results revealed that emotional awareness and regulation jointly mediate the relationship between self-esteem and SWB.

Computational mediation models increasingly incorporate neural and linguistic signals. For instance, transformer-based architectures have been utilized in Fisher et al. (2025) to analyze adolescents' online writing, identifying linguistic markers of emotion regulation difficulties. Such models provide interpretable mediators that can inform both digital assessments and targeted interventions.

### 4.3 Human-AI collaboration for adolescent support

While computational models excel in pattern recognition, they must integrate with human expertise to achieve ethical and context-aware applications. It has been argued in Sun et al. (2025) that “computational psychiatry” should prioritize human-AI synergy, where algorithms augment rather than replace counselors and educators. Similarly, a hybrid intervention has been proposed in Wahyunil et al. (2024) combining mindfulness-based digital modules with counselor-guided reflection, improving self-regulation and resilience.

### 4.4 Synthesis and implications

As shown in Table 3, collectively these developments underscore the potential of computational frameworks to personalize emotional and educational interventions. Yet, challenges persist concerning data privacy and algorithmic bias (Sun et al., 2025; Abro et al., 2014). Future research should integrate explainable AI methods and cross-cultural validation to ensure equity and transparency. The synergy of affective computing (Picard, 1997) and positive psychology (Seligman, 2011) can help translate digital signals into actionable insights that support holistic adolescent development.

**Table 3:** Representative computational studies addressing ER and SWB.

References	Methodology	Data type	Key findings	Domain
Jiang et al. (2021)	Random forest, SEM	Coping styles, surveys	Emotion regulation mediates coping and SWB; ML improves predictive power	Psychology/AI
Wenzel et al. (2022)	DSEM modeling	EMA, mood diaries	Daily emotion variability predicts SWB changes	Behavioral science
Abro et al. (2014)	SEM-ML integration	Speech, text, surveys	Emotion regulation mediates self-esteem-SWB link	Affective computing
Fisher et al. (2025)	Transformers, NLP	Online writings	Linguistic markers of poor regulation identified	Computational psychiatry
Wahyunil et al. (2024)	Hybrid digital + counseling	Mindfulness app data	Increased resilience and self-regulation	Educational psychology
Sun et al. (2025)	Human-AI collaboration framework	Conceptual	Ethical and collaborative design principles for computational psychiatry	Human-AI interaction

The computational literature extends these behavioral findings by demonstrating the feasibility of extracting and modeling psychological and contextual signals from increasingly diverse data sources. However, the evidence remains methodologically heterogeneous. Machine-learning studies often prioritize predictive performance, whereas SEM-based and longitudinal approaches provide greater emphasis on theoretically specified relationships and temporal processes. These approaches therefore offer complementary rather than interchangeable forms of evidence. A further limitation is that computational models may achieve useful predictive accuracy without necessarily clarifying the psychological mechanisms underlying the observed associations. This distinction reinforces the need for computational approaches to remain grounded in theoretically defined constructs and interpretable behavioral measures.

Importantly, computational approaches in this framework are not considered independently of psychological theory. Rather, psychological constructs provide the conceptual basis for defining the variables and relationships examined computationally. Self-esteem, ER, and SWB can therefore be represented through validated behavioral measures and contextual indicators and subsequently examined using statistical learning or longitudinal computational approaches. In this sense, computational methods extend psychological inquiry by allowing theoretically informed constructs to be examined across larger datasets and across multiple sources of information. Conversely, computational findings can help identify temporal or nonlinear patterns that may refine existing psychological models. The proposed integration therefore represents a bidirectional relationship between psychological theory and computational analysis rather than the replacement of theory-driven assessment by artificial intelligence.

## 5 Empirical evidence and trends

Empirical research over the past decade demonstrates the intertwined nature of ER, self-esteem and SWB among adolescents. While behavioral studies have traditionally emphasized psychometric and contextual determinants, computational research increasingly utilizes data-driven modeling to capture dynamic emotional processes. This section synthesizes evidence from both perspectives and highlights the emerging convergence between behavioral and computational insights.

### 5.1 Behavioral and psychological evidence

Behavioral studies consistently identify ER as a key mediator between self-related constructs and wellbeing. Self-compassion has been shown to indirectly enhance SWB through adaptive ER strategies, such as cognitive reappraisal, among high school students (Ge et al., 2019). Similarly, self-esteem and family cohesion have been examined in Andreja et al. (2017), and it has been reported that perceived social support amplifies positive affect and resilience in adolescents. Moreover, it has been found in Agustiningsih and Karimah (2023) that higher EI predicts greater life satisfaction and fewer depressive symptoms, supporting the mediating role of emotion regulation.

Cross-cultural studies reinforce these trends. ER profiles of Chinese and Western adolescents have been covered in Song et al. (2024), identifying culture-specific variations in suppression and reappraisal patterns. It is also important to note that collectivist orientations tend to buffer negative emotions via interdependent coping mechanisms, extending the contextual validity of ER theory. These results confirm earlier models Gross (1998), Gratz and Roemer (2004) and highlight the stability of ER-SWB relationships across cultural settings.

### 5.2 Computational and data-driven trends

The computational domain builds on behavioral foundations by leveraging multimodal data and machine learning to predict emotional outcomes. A multimodal neural framework combining physiological, textual and behavioral data has been developed to predict adolescents' momentary affect with 85% accuracy (Khoo et al., 2024). Likewise, it has been demonstrated in Saeed and Cha (2025) that deep affective models outperform classical psychometric prediction by incorporating linguistic and temporal cues from social media.

Longitudinal computational studies, such as Fisher et al. (2025) and Abro et al. (2014), confirm that ER mediates self-esteem-SWB relationships even in high-dimensional, multimodal contexts. However, empirical gaps remain concerning generalizability, interpretability and the ethical deployment of adolescent data (Sun et al., 2025).

### 5.3 Converging insights and empirical gaps

Behavioral and computational research increasingly converge toward a shared goal, i.e., understanding the mechanisms linking ER and SWB to support adolescent mental health. As illustrated in Figure 4, this convergence can be conceptualized as an interactive loop between theory-driven and data-driven approaches. Behavioral studies contribute the theoretical grounding, validated psychometric instruments and contextual understanding of ER, while computational approaches provide scalability, precision, and continuous monitoring through multimodal data.

**Figure 4:**
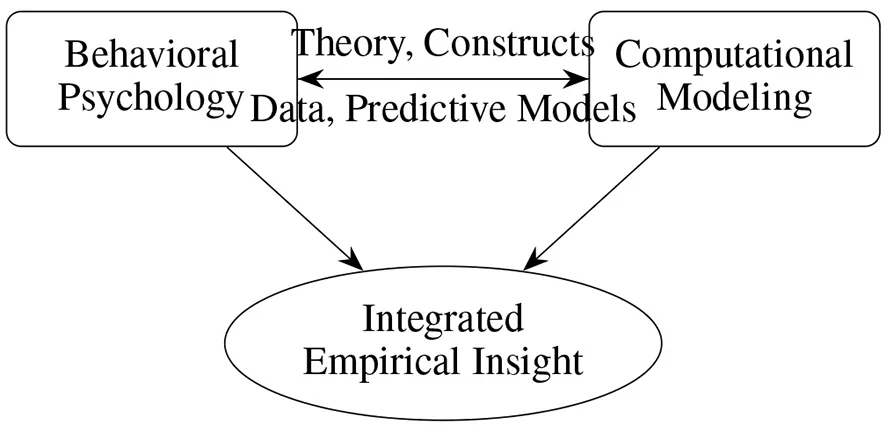
Convergence between behavioral and computational research on ER and SWB in adolescents.

Figure 4 visually depicts this interplay, showing how insights flow bidirectionally between behavioral psychology and computational modeling, ultimately integrating into actionable empirical frameworks. Such integration fosters the development of hybrid models that combine the explanatory depth of behavioral theories with the predictive power of computational systems.

Across the empirical literature, several broad trends emerge. Firstly, behavioral studies provide relatively consistent evidence for associations among ER, self-evaluative processes, and wellbeing, although the strength and direction of these relationships vary across samples and contexts. Secondly, computational studies demonstrate the potential to capture behavioral and contextual signals that are difficult to obtain through conventional assessments alone. Thirdly, the two studies differ in their evidentiary strengths: behavioral research offers stronger theoretical grounding and construct validity, whereas computational approaches offer scalability and the potential to model complex patterns. At the same time, neither perspective alone adequately captures the dynamic and context-dependent nature of adolescent wellbeing. These complementary strengths provide a rationale for an integrative framework rather than privileging either behavioral or computational approaches.

## 6 Discussion and future directions

Building upon the preceding sections, the reviewed literature reveals distinct yet interrelated contributions emerging from behavioral psychology, computational modeling and integrative frameworks in adolescent emotion research. To synthesize these insights, Table 4 summarizes the key thematic domains, representative studies, methodological approaches and implications for future research and practice. As shown, behavioral investigations emphasize the mediating role of emotion regulation and self-esteem in SWB, whereas computational approaches introduce scalable methods for continuous monitoring and predictive modeling. Empirical and integrative frameworks increasingly bridge these paradigms, offering ethically grounded, culturally responsive and data-informed strategies for supporting adolescent mental health.

**Table 4:** Summary of contributions and insights across behavioral, computational, empirical and integrative domains.

Domain	Key focus	References	Methodological features	Implications for research and practice
Behavioral psychology	Emotion regulation, self-esteem, and SWB relationships	(Ge et al., 2019; Andreja et al., 2017; Agustiningsih and Karimah, 2023)	Cross-sectional surveys, validated scales (ERQ, DERS, SWLS), SEM mediation models	Highlights ER as a mediator; cultural and contextual factors shape adolescent wellbeing
Computational modeling	Data-driven prediction of emotion and wellbeing	(Jiang et al., 2021; Abro et al., 2014; Khoo et al., 2024; Saeed and Cha, 2025)	Machine learning (RF, CNN–RNN), SEM–ML pipelines, digital phenotyping, multimodal data fusion	Provides scalable and continuous monitoring; advances precision in mental health modeling
Empirical trends	Convergence of behavioral and computational evidence	(Song et al., 2024)	Mixed-method and longitudinal approaches, cross-cultural and digital datasets	Supports hybrid, theory-informed computational frameworks for adolescent ER and SWB
Integrative framework	Unified, ethical, and adaptive ecosystem for adolescent wellbeing	(Sun et al., 2025; Seligman, 2011; Salazar and Warren, 2024)	Interdisciplinary design combining behavioral constructs, AI-based models, and feedback loops	Promotes explainable, culturally inclusive, and ethically grounded interventions in education

The evidence demonstrates strong convergence between theory-driven and data-driven paradigms, signaling a shift toward integrative, technology-supported behavioral science. This section synthesizes key insights, outlines practical and theoretical implications and identifies future research trajectories.

### 6.1 Integrating behavioral and computational insights

Behavioral models provide the theoretical foundation for understanding the cognitive and emotional mechanisms underlying adolescent wellbeing, emphasizing constructs, such as self-esteem, self-compassion and EI (Ge et al., 2019; Agustiningsih and Karimah, 2023). Computational models complement these frameworks by offering quantitative tools for dynamic prediction and personalized intervention (Jiang et al., 2021; Abro et al., 2014; Khoo et al., 2024).

Building on the psychological mediation model illustrated in Figure 1, the broader framework proposed in this paper integrates behavioral constructs, computational modeling and adaptive feedback. Thus, while Figure 1 captures a specific psychological relationship identified in the literature, Figure 5 represents the overarching behavioral-computational framework of the manuscript. Behavioral data, psychometric scales and contextual factors feed into computational systems that use multimodal data analytics, machine learning and digital phenotyping to detect emotional states and predict SWB. These computational outputs, when interpreted through behavioral theory, inform adaptive and ethically grounded interventions. The result is a feedback-oriented ecosystem that unites theory validation and real-time monitoring.

**Figure 5:**
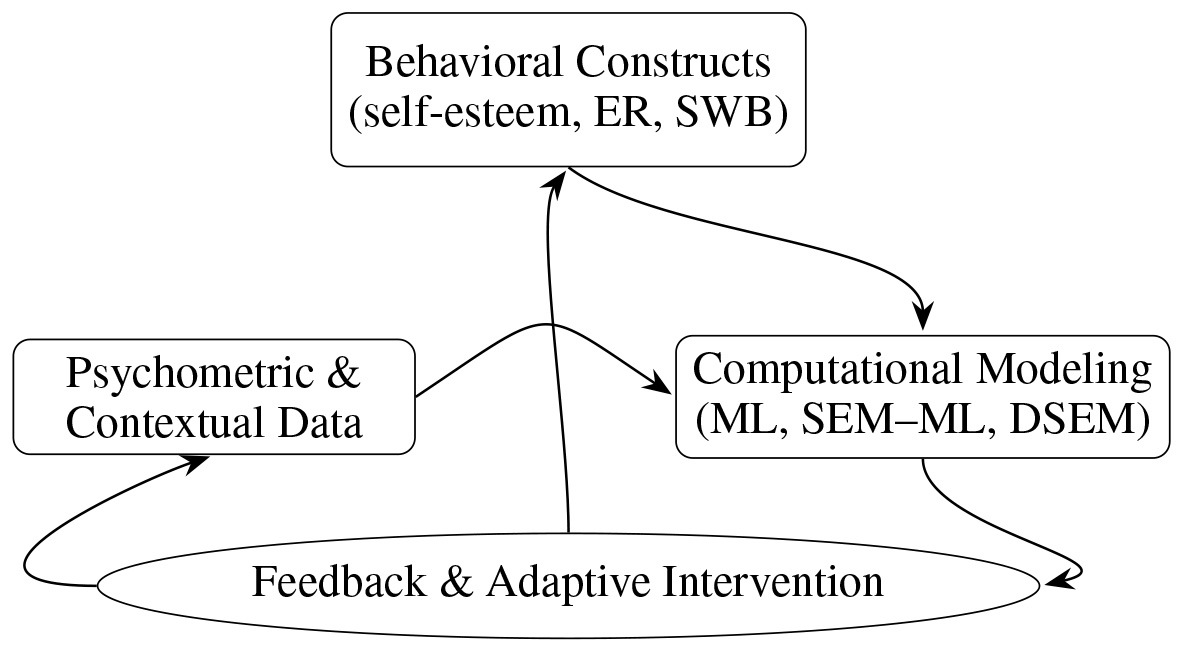
An integrative framework linking behavioral constructs, computational modeling, and adaptive feedback for adolescent wellbeing.

As illustrated in Figure 5, the framework links psychological constructs to computational analysis by treating behavioral and contextual measures as theoretically informed inputs to computational models. Whereas it also allows computational outputs to inform subsequent interpretation and adaptive feedback.

This integration allows research to move beyond static self-reports toward dynamic, continuous and personalized monitoring. For instance, digital phenotyping through smartphones can complement self-report scales like the ERQ or SWLS by identifying momentary affective shifts (Salazar and Warren, 2024). Such hybrid approaches may advance precision mental health and preventive education.

A practical implementation of this framework can be illustrated through a hypothetical longitudinal study of high school students. A research team could first collect baseline measures of self-esteem, ER and SWB using validated psychological scales. Students could then complete brief weekly or daily assessments of mood and contextual experiences through a secure digital platform, which is supplemented by non-invasive behavioral or physiological measures. This data could be analyzed using statistical models to examine theoretically specified relationships, while machine learning models could be used to identify patterns associated with changes in wellbeing over time. The two streams of analysis would then be compared to determine whether computationally identified patterns are consistent with established psychological relationships. Importantly, the resulting predictions would not be treated as diagnoses. Instead, they could be used to identify periods or contexts in which additional support may be appropriate, with interpretation and intervention remaining under the supervision of trained educators or counselors. This example illustrates how behavioral theory and computational analysis can operate as complementary components of a single research process rather than as separate methodological approaches.

### 6.2 Practical and ethical considerations

As computational approaches become increasingly embedded in educational and psychological practice, researchers must address both the practical and ethical dimensions of integrating AI into ER and wellbeing interventions for adolescents. The literature indicates that while AI tools, ranging from emotion-aware chatbots to adaptive learning platforms, offer scalable opportunities for emotional support and self-regulated learning, they also pose significant developmental and ethical challenges (Garcia et al., 2025; Aziz et al., 2026; Klimova and Pikhart, 2025).

#### 6.2.1 Balancing risks

AI applications in education have shown promise in enhancing student motivation and socio-emotional engagement. Studies demonstrate that conversational agents and digital wellbeing platforms can alleviate stress and improve emotional awareness among high school students (Samatha et al., 2025; Romo Aréchiga et al., 2024; Kavitha et al., 2024). However, over reliance on AI systems may desensitize emotional learning and impede authentic human interaction–core components of EI and long-term SWB (Keshishi and Hack, 2023; Crawford, 2021; Changoluisa Jaya, 2024). Excessive automation risks creating an environment of algorithmic dependency, where critical thinking and interpersonal empathy are known to decline over time (Deckker and Sumanasekara, 2025; Yang, 2022).

#### 6.2.2 Ethical and privacy implications

A recurring theme across recent reviews is the tension between personalization and privacy. AI-driven models rely heavily on sensitive emotional and behavioral data to tailor interventions, raising ethical questions about consent, data ownership, and emotional surveillance (Fuszek et al., 2025; Gupta, 2025). Algorithmic bias (Sun et al., 2025) further threatens to reinforce social inequities, especially when emotion-recognition systems are trained on culturally narrow datasets. Scholars emphasize the need for transparent algorithms, ethical data handling and interdisciplinary oversight committees that ensure the protection of adolescent users (Vieriu and Petrea, 2025; Tang et al., 2023; Konadu and Kusi, 2025).

#### 6.2.3 Human-AI collaboration and emotional authenticity

Emerging frameworks advocate for hybrid approaches that position AI as an assistive partner rather than a replacement for human educators or counselors. Conversational AI tools can provide immediate support but maintaining emotional authenticity and empathy requires a human-in-the-loop model that preserves relational depth (Aziz et al., 2026; Adesina et al., 2024). This perspective aligns with the *whole-child* approach to education, which underscores the need for emotionally responsive, ethically grounded and culturally inclusive AI design (Garcia et al., 2025; Yang, 2022). Such models must consider developmental sensitivity, ensuring that technology complements rather than dominates adolescents' emotional and cognitive growth trajectories.

Collectively, the literature calls for a shift toward human-centered, ethically aligned AI integration in education. This includes developing cross-disciplinary guidelines for data governance and emotional inclusivity. Future systems should employ participatory design principles that involve students, educators, parents and mental health professionals in shaping ethical boundaries and ensuring that AI remains a facilitator of wellbeing rather than a substitute for authentic human care. As it has been argued in Fuszek et al. (2025), ethical frameworks must evolve alongside technological capabilities, cultivating an ecosystem where computational precision reinforces rather than compromises emotional resilience and SWB in adolescents.

### 6.3 Future research directions

Future studies should extend current interdisciplinary insights by advancing both the methodological and ethical foundations of computational research on adolescent wellbeing. One promising avenue involves the development of multimodal longitudinal models that combine behavioral surveys and physiological sensors to capture the dynamics of emotion regulation over time. Such models would enable researchers to analyze how affective and contextual factors evolve in real-world settings, offering richer and more temporally sensitive insights than cross-sectional surveys alone. Integrating wearable data with ecological momentary assessment (EMA) and self-report measures can provide a multidimensional understanding of how emotional states fluctuate throughout the school year and across different stressors.

Another emerging direction involves the advancement of explainable artificial intelligence (XAI) for mental health research. While machine learning models have shown promise in predicting emotional wellbeing, the interpretability of such models remains a critical challenge. Future research should focus on transparent architectures that visualize how features of ER and contextual factors contribute to predictions of SWB. Techniques, such as attention visualization and feature attribution, can make AI-driven mental health tools more interpretable to educators and policy-makers, thereby fostering trust and ethical accountability in real-world applications.

Cross-cultural adaptation also represents a key frontier in the computational psychology of adolescent wellbeing. Many existing predictive frameworks have been developed using data from Western educational systems, which may not generalize to other sociocultural contexts. Researchers should therefore validate computational models across diverse populations to ensure inclusivity and cultural fairness (Song et al., 2024). Comparative studies across different linguistic and cultural settings can help in identifying both universal and culture-specific mechanisms of ER and SWB, thereby enriching global perspectives on youth mental health.

Personalized feedback systems offer another powerful opportunity for advancing applied research. JITAIs can deliver context-sensitive emotional support, guiding students toward healthier regulation strategies as they navigate academic and social stressors. These systems, when grounded in affective computing and digital phenotyping frameworks, can enhance self-awareness and resilience by providing timely and personalized prompts that adapt to users' changing emotional states. However, ensuring that such systems remain ethically aligned, while respecting privacy and autonomy, will be essential for their sustainable deployment.

Finally, there is an urgent need to bridge computational research with educational policy and practice. Insights from affective modeling and digital wellbeing analytics can inform evidence-based curricula that promote positive education and emotional resilience among students (Seligman, 2011). Policymakers and educators should work collaboratively with data scientists and psychologists to translate algorithmic findings into practical frameworks that nurture EI and wellbeing in schools. Integrating computational evidence into educational reform can ensure that AI-enhanced learning environments remain human-centered and supportive of holistic adolescent development.

Together, these research directions can advance an evidence-based and technologically supported paradigm for adolescent mental health and wellbeing. By integrating computational precision with human empathy, future studies have the potential to not only predict but also meaningfully enhance the emotional lives of young people in a rapidly digitizing world.

## 7 Conclusion

This integrative review examined the intertwined dimensions of ER and SWB in high school students by integrating insights from behavioral psychology and computational sciences. Behavioral studies elucidate the cognitive and affective mechanisms underlying adolescents' self-regulation and mental wellness, while computational approaches offer scalable, data-rich frameworks for continuous monitoring and predictive modeling. Empirical trends suggest a clear convergence, i.e., theory-driven measures of emotion and wellbeing are now complemented by digital and physiological analytics, yielding a more holistic picture of adolescent mental health. However, significant gaps persist in longitudinal modeling, ethical AI integration and cultural adaptation of computational tools. Future research should thus pursue cross-disciplinary collaborations that unite psychologists and educators to develop interpretable, ethically grounded and culturally responsive systems for adolescent mental health support. By bridging behavioral validity and computational precision, such efforts can pave the way toward truly personalized wellbeing interventions for youth in educational and clinical contexts.

Although the proposed framework is motivated by evidence from adolescent populations, its conceptual structure is sufficiently general to be adapted and validated for other developmental stages, including emerging adulthood and older adult populations.

The central contribution of this integrated review is an integrative framework that connects behavioral science and computational approaches for understanding adolescent emotion regulation and SWB. By placing psychological constructs and empirical evidence at the foundation, the framework provides a structured pathway for incorporating computational modeling, multimodal data, and adaptive feedback without separating these approaches from their theoretical and contextual foundations. Future research can build on this framework through longitudinal and culturally diverse studies while maintaining attention to interpretability, ethical safeguards, and human oversight.

Based on the evidence reviewed, three priorities appear particularly important for advancing interdisciplinary research on adolescent mental health. Firstly, future studies should prioritize longitudinal and multimodal designs that combine behavioral assessments with contextual and digital data to examine how ER, self-esteem, and SWB change over time. Secondly, computational models should remain closely grounded in psychological theory and should emphasize interpretability, allowing computational findings to be meaningfully related to established psychological constructs and mechanisms. Thirdly, future work should investigate ethically grounded and culturally sensitive human–AI approaches in which computational tools complement, rather than replace, professional and interpersonal support. Together, these priorities can help move the field from predominantly cross-sectional and descriptive evidence toward theoretically informed and context-sensitive models of adolescent wellbeing.
